# Effects of message framing, psychological distance, and risk perception on exercise attitude in Chinese adolescents

**DOI:** 10.3389/fped.2022.991419

**Published:** 2022-08-30

**Authors:** Xiaohua Wang, Xiyan Duan, Shichen Li, Te Bu

**Affiliations:** ^1^School of Physical Education and Health, Wenzhou University, Wenzhou, China; ^2^College of Physical Education, Hunan Normal University, Changsha, China

**Keywords:** physical activity, obesity, temporal distance, health communication, health promotion

## Abstract

**Objectives:**

Health communication campaign that promotes physical activity may aid in reducing the prevalence of childhood and adolescent obesity in China. This study examined the effects of message framing and psychological distance on the exercise attitude of Chinese adolescents, along with the interactive role of risk perception.

**Methods:**

Participants in this study were between 10 and 19 years of age. Three experiments were conducted independently. In experiment 1, 76 participants were recruited to assess the effectiveness of message manipulation. In experiment 2, 40 participants were recruited to compare the effects of gain- and loss-framed messages on the exercise attitude. In experiment 3, 37 participants were recruited to explore the interaction between message framing (gain vs. loss), temporal distance (proximal vs. distal), and risk perception (low vs. high) on the exercise attitude. Exercise attitude and risk perception were assessed by Likert-type questionnaires.

**Results:**

There was a significant main effect of message framing (*P* < 0.05), with the gain-framed message inducing a stronger exercise attitude than the loss-framed message. There was a significant main effect of temporal distance (*P* < 0.05), with the proximal temporal distance condition inducing a stronger exercise attitude than the distal temporal distance condition. In addition, a significant interaction (*P* < 0.05) was observed, and the perception of obesity-related risk was a crucial moderator of the message framing and temporal distance. Regardless of whether they were exposed to a gain- or loss-framed message, the proximal temporal distance condition induced a stronger exercise attitude in participants with a high risk perception (*P* < 0.05).

**Conclusion:**

Messages promoting exercise that are framed as having a near-future gain effect and that emphasize disease risks are effective in motivating adolescents to engage in physical activity.

## Introduction

Obesity is caused by an excess of energy intake over energy expenditure, which develops gradually over time and results in an excessive accumulation of body fat. Obesity leads to a wide array of health problems, including hypertension, type 2 diabetes, coronary heart disease, osteoarthritis, and stroke, and contributes significantly to morbidity and mortality (1). The Chinese government prioritizes preventing and resolving the issue of childhood obesity. In 2016, the Outline of the “Healthy China 2030” Plan (2) proposed strengthening the prevention and treatment of childhood overweight and obesity. According to the most recent Report on Chinese Residents' Chronic Diseases and Nutrition 2020, the prevalence of overweight and obesity in school-aged children aged 6–17 has reached 19% (3). Lifestyle modifications such as dietary intervention and physical activity are necessary for the effective management of obesity (1). In response to the alarming obesity trend, the National Fitness Program (2021–2025) emphasizes the promotion of physical activity for key populations and exercise interventions for childhood obesity (4). Participation in physical activity is voluntary and, thus, subject to individual's decision-making processes. Correspondingly, it is crucial to help children and adolescents shape a positive exercise attitude first.

According to the theory of reasoned action, behavioral attitude and subjective norms influence behavioral intention, and behavioral intention can directly determine the occurrence of behavior (5). The exercise attitude-behavior model has subsequently been proposed for exercise promotion (6). Theoretically, an individual's exercise attitude exerts positive influence on their exercise intention, which in turn influences their actual exercise behavior. With the continuous development and improvement of research theories pertaining to exercise attitude, more studies have been conducted on the practical applications of exercise attitude. Qiu and Zhang (7) reported a single session of cognitive learning can successfully modify implicit exercise attitude; nevertheless, its effect on physical activity is very transient, lasting only 1 day in their study. Liu and Li demonstrated that the exercise attitude of Chinese University students positively predicted exercise behavior, including duration, intensity, and frequency of exercise (8). A longitudinal study revealed that American adolescents with more positive exercise attitude engaged in 30–40% more moderate-to-vigorous physical activity at 5-year and 10-year follow-up than those with less positive exercise attitude (9). Recently, French researchers looked into the effect of eHealth (i.e., online and social media exercise and physical activity promotion) on exercise attitude and actual exercise activity during the first COVID-19 pandemic wave (10). eHealth users demonstrated significantly higher levels of exercise attitude, vigorous physical activity, and total physical activity per week compared to non-users. These findings illustrate the importance of developing health communication with evidence-based material for encouraging an exercise-friendly attitude, and its therapeutic implications are now being explored and expanded (11).

Message framing is a persuasive message that can influence an individual's decision preferences by altering the presentation of the alternative message (12). There are three types of framing effect: risky choice framing, attribute framing, and goal framing (13). The goal framing effect, which describes whether a persuasive message emphasizes the positive outcomes of performing an act or the negative outcomes of not performing the act, has gained popularity in health communication research of exercise attitude. In general, the effect associated with goal framing is both positive and diverse. Latimer et al. (14) presented gain- or loss-framed messages to sedentary, healthy participants at baseline, week 1, and week 5. At week 9, it was found that gain-framed messages led to greater participation in physical activity than loss-framed messages. Similarly, Berenbaum and Latimer-Cheung found that gain-framed messages resulted in stronger exercise attitude than loss-framed messages when presented to female University students (15). In anther study, University students who received framed mental health messages on social media had a stronger exercise attitude than those who got random health facts unrelated to physical activity (16). However, the authors did not find a significant difference between gain- and loss-framed messages in terms of exercise attitude or actual exercise behavior as measured by total MET minutes per week. This disparity in outcomes may be attributable to the heterogeneity of the focus participants investigated. For instance, Song et al. (17) demonstrated that exercise self-efficacy was negatively affected when individuals with high body image dissatisfaction viewed an image of themselves on a video game screen, indicating that overweight and obese individuals may have different psychological responses to physical activity. In addition, existing research have not explored the exercise attitude of children and adolescents in a more homogeneous manner. In light of these gaps, research of message framing should explore the framing effect on specific populations and likely include perception of disease risks as a covariate.

When constructing health communication aimed at fostering a more positive exercise attitude, it is crucial to take into account variables that mediate the presence, strength, or absence of framing effect. First, the relative persuasiveness of message framing is dependant on the psychological distance (18). Psychological distance is a cognitive separation between the self and other things, such as other people, events, or times (19). The theory posits four dimensions of psychological distance, including spatial distance, temporal distance, social distance, and hypotheticality. Research on marketing communication suggests that consumers in a pre-decisional mindset are more likely to be persuaded by psychologically distal messages, whereas those in a post-decisional mindset are more likely to be persuaded by psychologically proximal messages (20). To date, very few studies have been conducted on the interaction between message framing and psychological distance on exercise attitude (21) and are limited to the general population. A recent study examined the effects of a 3-month exercise promotion program on the attitude and behavior of 18- to 65-year-old adults (22). It has been observed that persons with more negative distal affective attitudes at baseline exhibited the highest proximal affective attitudes toward daily exercise. Moreover, the specific effect of temporal distance in adolescents is completely undocumented.

Second, the relative persuasiveness of message framing is also influenced by the risk perception associated with the advocated health behavior (23). Risk perception is an individual's subjective, more intuitive evaluation of risk (24). Research on health communication found that perception of junk food risk can amplify the relationship between regulatory focus and health consciousness, and message framing combined with the appropriate regulatory focus has a positive effect on consumers' intentions to avoid junk food (25). Similarly, a perception of obesity risk, may also influence one's exercise attitude, which has not previously been noted in published literature. Considering the cognitive connection between the perceived risk and behavioral responses in health decision-making, it is essential to understand the role of risk perception in shaping exercise attitude among adolescents in order to implement effective message framing.

Integrating health promotion campaigns into the school curriculum and daily life may be a promising strategy for encouraging children and adolescents to improve their health-related beliefs and behaviors. To develop tailored exercise promotion, a comprehensive understanding of the mechanisms underlying the formation of exercise attitude is urgently required. This study therefore examined the interaction between message framing, temporal distance, and risk perception on the exercise attitude of Chinese adolescents.

## Experimental 1

### Methods

#### Participants

Experiment 1 was designed to validate the framed messages employed in experiments 2 and 3. Participants had not previously participated in similar experiments. Seventy six students participated in experiment 1, with 39 participants being female. Their age was 14.5 ± 1.2 yrs (girls, 14.4 ± 1.2 yrs; boys, 14.7 ± 1.1 yrs). All participants and their legal guardians were verbally and in writing informed about the research prior to providing written informed consent to participate. This experiment was approved by the Ethical Review Board of Hunan Normal University.

#### Instrument

Table 1 presents the messages framed to motivate exercise. The gain-framed message emphasized the advantages of regular participation in exercise and sports, whereas the loss-framed message underlined the disadvantages of non-participation.

**Table 1:** Framed messages (translated from Chinese).

**Gain-framed message**	**Loss-framed message**
According to the “Core information and interpretation of Chinese youth health education (2018 version)” published by the National Health Commission, if you regularly engage in scientific exercise and sports, you will burn more calories and your body will become healthier and slimmer. In addition, you will feel more comfortable during your exercise and sports and grow to appreciate it more and more. And the cardiovascular disease, fatty liver disease, and other health problems caused by obesity will gradually disappear as exercise habit is maintained. When you reduce the trouble caused by obesity, you will be more appealing to your peers.	According to the “Core information and interpretation of Chinese youth health education (2018 version)” published by the National Health Commission, if you do not engage in regular exercise and sports, you run the risk of gaining weight and your physical health will deteriorate. In addition, subsequent exercise and sports will be increasingly difficult, and you will develop an increasing aversion to it. And as a result of obesity, cardiovascular disease, fatty liver disease, and other health conditions will worsen if you do not exercise. Furthermore, social rejection from your peers will increase as a result of your obesity.

The effectiveness of framing manipulation was evaluated based on participants' affective reaction and information acceptance to the framed message. Affective reaction to the framed message was measured using a peer-reviewed, 7-point semantic differential scale (scale: 1, mostly negative; 7, mostly positive) (26). It was anticipated that the gain-framed message would produce stronger positive affect, whereas the loss-framed message would produce stronger negative affect.

Information acceptance of the framed message was measured using a peer-reviewed, 7-point semantic differential scale (27). Participants rated the extent to which they thought the message was relevance (1, very relevant; 7, not at all relevant), interest (1, very interesting; 7, not at all interesting), objectivity (1, very objective; 7, not at all objective), and exaggeration (1, very exaggerated; 7, not at all exaggerated). In addition, participants rated their level of agreement with the message (1, I totally agree; 7, I totally disagree). The information acceptance score was calculated by reversing (except “exaggerated”), combining, and averaging all individual item scores. It was anticipated that the information acceptance score of both framed messages would be higher than the median score 4. Cronbach alpha of this scale was 0.81 (27).

#### Procedure

Participants read gain- and loss-framed messages at random. They completed the affective reaction scale after reading each message and the information acceptance scale after reading both messages.

#### Statistics

The deidentified data from experiments 1, 2, and 3 that supporting the conclusions of this study are available on figshare (https://doi.org/10.6084/m9.figshare.20286075.v2). Data were analyzed using the IBM SPSS Statistics (version 26.0). Data are expressed as mean ± standard deviation. A two-tailed Welch's *t*-test (28) was used to compare the affective reaction score between the gain- and loss-framed messages. A one-sample Wilcoxon signed rank test was used to compare the information acceptance score against the median score 4. A *P*-value <0.05 indicates statistical significance.

### Results

Affective reaction scores of gain- and loss-framed messages were compared. On average, gain-framed massage (6.12 ± 0.71) was perceived significantly more positive than loss-framed message (2.76 ± 1.30), *t*_(116.5)_ = 19.80, *P* < 0.001. The medium score for information acceptance was 5.4, which is significantly higher than 4, *P* < 0.001. In summary, the manipulation check confirmed that both framed messages satisfy the experimental conditions.

## Experimental 2

### Methods

#### Participants

Experiment 2 compared the framing effect on the exercise attitude. According to previous research, it was hypothesized that the gain-framed message should be more persuasive than the loss-framed message. Based on the G^*^POWER (version 3.1), a total of 34 participants were required to ensure a medium effect size (statistical power = 0.8, effect size = 0.25, significance level = 0.05). Forty students participated in experiment 2, with 23 participants being female. Their age and figure rating scale (29) were 14.4 ± 1.2 yrs (girls, 14.2 ± 1.2 yrs; boys, 14.7 ± 1.1 yrs) and 5.0 ± 1.0, respectively. All participants and their legal guardians were verbally and in writing informed about the research prior to providing written informed consent to participate. This experiment was approved by the Ethical Review Board of Hunan Normal University.

#### Instrument

The messages framed to influence exercise attitude are identical to those used in experiment 1. Exercise attitude was measured using a peer-reviewed, 7-point semantic differential scale (15). The questionnaire was constructed as “I consider routine scientific exercise to be” followed by seven adjective pairs with the following anchors: “bad–good; beneficial–harmful (reverse coded); worthless–valuable; unenjoyable–enjoyable; pleasant–unpleasant (reverse coded); interesting–boring (reverse coded); relaxing–stressful (reverse coded)”. The average score of seven items reflects an individual's exercise attitude, with a higher score indicating a greater propensity for exercise participation. Cronbach alpha of this questionnaire was 0.76–0.78 (15).

#### Procedure

Participants chose images that resembled their own body shape based on the figure rating scale (29). Participants then read gain- and loss-framed messages at random. After reading each message, participants rated their exercise attitude and took a 5-min break between messages.

#### Statistics

Data were analyzed using the IBM SPSS Statistics (version 26.0). Data are expressed as mean ± standard deviation. A one-tailed Welch's *t*-test was used to compare the exercise attitude score between the gain- and loss-framed messages. A *P*-value <0.05 indicates statistical significance.

### Results

Participants scored significantly higher on the exercise attitude after reading the gain-framed message (5.78 ± 0.79) than after reading the loss-framed message (5.28 ± 1.31), *t*_(64.29)_ = 2.076, *P* = 0.02.

## Experimental 3

### Methods

#### Participants

Experiment 3 examined the effects of message framing and temporal distance on the exercise attitude of students who perceived a high risk of obesity and those who perceived a low risk of obesity. Based on the G^*^POWER (version 3.1), a total of 34 participants were required to ensure a medium effect size (statistical power = 0.8, effect size = 0.25, significance level = 0.05). Thirty seven students participated in experiment 3, with 24 participants being female. Their age and figure rating scale (29) were 14.3 ± 1.7 yrs (girls, 14.3 ± 1.7 yrs; boys, 14.3 ± 1.7 yrs) and 6.5 ± 1.6, respectively. All participants and their legal guardians were verbally and in writing informed about the research prior to providing written informed consent to participate. This experiment was approved by the Ethical Review Board of Hunan Normal University.

#### Instrument

Risk perception was assessed with items adapted from the risk behavior diagnosis scale (5-point Likert scale: 1, strongly disagree; 5, strongly agree) (30). Each of the two dimensions of risk, severity and susceptibility, consists of three items. Severity was measured using three items, including “I consider my obesity to be severe”, “I believe that the health consequences of obesity are severe”, and “I believe that the consequences of obesity due to a lack of regular exercise are significant”. Susceptibility was measured using three items, including “I am experiencing the risks of being obese”, “I am highly susceptible to the dangers associated with obesity”, and “I am likely to experience the risks associated with being overweight”. The average score of six items indicates an individual's perception of obesity, with a higher score indicating a greater risk.

The message framing used in experiment 3 were identical to those used in experiment 1, with the addition of a temporal distance factor for the text material of exercise attitude. In this study, temporal distance refers to the period of time between when obese middle school students participated in physical activity and the present day. The manipulation of temporal distance is based on the research of Trope and Liberman (31), in which the proximal temporal distance is expressed as “tomorrow” and the distal temporal distance as “a year from now”. The questionnaire of exercise attitude was altered as follows: “I believe that beginning tomorrow, routine scientific exercise is…” and “A year from now, I anticipate routine scientific exercise to be…”.

#### Procedure

Before message exposure, participants' risk perception was evaluated; those with a risk perception ≥3 were considered to be high risk of obesity, while those with a risk perception <3 were considered to be low risk of obesity. Participants chose images that resembled their own body shape based on the figure rating scale (29). Participants then randomly read four different categories of message: gain-proximal, gain-distal, gain-proximal, and loss-distal. After reading each category of message, participants completed the exercise attitude scale and took a 5-min break for each category completed.

#### Statistics

Data were analyzed using the IBM SPSS Statistics (version 26.0). Data are expressed as mean ± standard deviation. The interaction effect on the exercise attitude was analyzed by repeated measures analysis of variance. Two within-subjects variables are message framing (gain vs. loss) and temporal distance (proximal vs. distal) and one between-subjects factor is perceived risk (low vs. high). A customized SPSS syntax employing LSD as post hoc was constructed to decompose a significant three-way interaction into simple main effects. A *P*-value <0.05 indicates statistical significance.

### Results

Initially, we examined the effects of message framing and temporal distance on the exercise attitude, without differentiating the risk perception levels among the participants. There was a significant main effect of message framing on the exercise attitude, [F_(1, 36)_ = 7.871, P = 0.008]. When participants read the gain-framed message (5.00 ± 1.25), they had stronger exercise attitude than when they read the loss-framed message (4.60 ± 1.34). There was a significant main effect of temporal distance on the exercise attitude, [F_(1, 36)_ = 13.416], P <0.001. Participants' exercise attitude was stronger in the proximal temporal distance condition (5.25 ± 0.98) than in the distal temporal distance condition (4.35 ± 1.44). The interaction between message framing and temporal distance was not significant, [F_(1, 36)_ = 1.671], P = 0.204.

Then, the between-subjects factor risk perception was added to investigate whether participants' exercise attitude differed when comparing low and high risk perception. A two-way interaction between message framing and risk perception was not significant, [F_(1, 35)_ = 1.627], P = 0.211, nor a two-way interaction between temporal distance and risk perception, [F_(1, 35)_ = 0.829], P = 0.369. However, there was a significant three-way interaction between message framing, temporal distance, and risk perception, [F_(1, 35)_ = 4.927], P = 0.033.

Subsequently, we performed a simple main effects analysis and the results are presented in Table 2. Participants with a low risk perception had stronger exercise attitude in the proximal psychological distance condition after reading the gain-framed message (*P* = 0.032). However, temporal distance had no effect on the exercise attitude of participants with a low risk perception in the reading loss-framed message (*P* = 0.132). Participants with high risk perception had stronger exercise attitude in the proximal temporal distance condition than in the distal temporal distance condition, regardless of whether they read the gain- or loss-framed message (all *P* < 0.05).

**Table 2:** Results of simple main effects.

**Risk perception**	**Message framing**	**Temporal distance**	**Exercise attitude**	***F***	***P***
Low	Gain	Proximal	5.64 ± 0.92	4.973	0.032
		Distal	4.88 ± 1.24		
	Loss	Proximal	4.99 ± 1.12	2.378	0.132
		Distal	4.38 ± 1.38		
High	Gain	Proximal	5.13 ± 0.84	5.155	0.029
		Distal	4.33 ± 1.58		
	Loss	Proximal	5.25 ± 0.97	13.193	<0.001
		Distal	3.78 ± 1.43		

Data are expressed as mean ± standard deviation.

## General discussion

Based on experimental results, the gain-framed message induced stronger exercise attitude than loss-framed message. In addition, under the proximal temporal distance condition, adolescents displayed stronger exercise attitude. Moreover, we demonstrate that the perception of obesity-related risk is a crucial moderator of the framing effect. For adolescents with a low risk perception, proximal temporal distance is more likely to induce the gain-framing effect. For adolescents with a high risk perception, homogeneity of risk perception is high, resulting in a feeling of temporal oppression. Consequently, proximal temporal distance condition induced stronger exercise attitude, regardless of whether adolescents were exposed to the gain- or loss-framed message. There are significant theoretical and practical implications for these findings.

In health communication literature, gain-framed messages are generally more effective than loss-framed message in promoting health promotion behaviors such as smoking cessation and physical activity (32), while loss-framed messages are more effective in promoting disease prevention behaviors (33). For example, Notthoff et al. (34) chose six exercises, and the advantages and disadvantages of these six exercises were presented in gain- and loss-framed messages, respectively. It was found that elderly individuals felt more motivated to engage in physical activity when gain-framed messages were paired with health promotion. Therefore, gain-framed messages are recommended for exercise behavioral interventions when designing exercise promotion campaigns (35). Based on the existing literature, we conducted a more comprehensive study on the exercise promotion strategies for obesity intervention.

We selected adolescents, a focus population for the prevention of overweight and obesity. Obesity in youth is a significant predictor of adult disease and premature mortality (36). The adolescents' exercise attitudes were significantly stronger after reading the gain-framed message than after reading the loss-framed message, corroborating previous research. In a broader context, exercise attitude is a health-related decision-making process that can be explained by the prospect theory. Prospect theory posits that in a benefit scenario, people tend to avoid risk, whereas in a loss scenario, they tend to seek risk (37). When adolescents read about the various health benefits of physical activity, they will develop positive attitudes toward physical activity. In contrast, when adolescents read that not exercising will result in a greater health burden, they may experience acute stress responses (38) and cause active avoidance behavior (39). Due to psychological stress, information that emphasizes the severe health consequences of inactivity may have an acute negative impact on their exercise attitude. In addition, the loss-framed message includes self-esteem-related language such as “social rejection from your peers will increase as a result of your obesity”, which may impede exercise self-efficacy (17) and should be avoided in the future. In light of our findings, we suggest information encouraging adolescent physical activity be framed in terms of health benefits and increased self-confidence.

In health communication research, psychological distance can affect an individual's comprehension of the message framing, thereby impacting his or her decision-making behavior (40). It has been demonstrated that temporally proximal framing makes risks appear more immediate and concrete than temporally distal framing, leading to increased self-risk perceptions, intentions to engage in precautionary behavior, concern and anxiety about the hazard, and risk communication effectiveness (41). However, there is a paucity of research on the effect of psychological distance on exercise behaviors, and the interaction between message framing and temporal distance remains unknown. We found that adolescents' exercise attitudes were consistently stronger in the proximal temporal distance condition than in the distal temporal distance condition, regardless of whether the messages were gain- or loss-framed. This consistency (as opposed to temporal variation) in the near future health decision-making can be explained by the construal level theory (31). In the presence of health stress, individuals are more eager to reduce their health burdens through physical activity, i.e., they anticipate more specific, detailed changes in their bodies as a result of physical activity in the proximal temporal distance condition. This type of cognitive preference for near future certainty has substantial implications for the creation of persuasive health promotion messages. The messages should emphasize the definitive, key, and immediate health outcomes of regular exercise participation. Consequently, the target population, such as obese adolescents, would view the desired health outcome as more attainable, and their decision status mindsets would assist them in decisively committing to near-future behavior modification.

Compared to everyday decisions, health decisions in response to risk perception are infrequent but of the utmost significance. Individual health attitudes and behaviors are influenced by the degree to which individuals perceive a threat to their health (42). When an individual is exposed to health information that is relevant to their current health concerns, their self-health awareness of disease threats increases, and this cognitive consciousness and acceptance motivates self-care behaviors. In addition, it has been suggested that proximal threat (in terms of temporal distance) may be more effective at motivating pro-health behavior (43). We observed this perception-attitude link, in which a high risk perception led to a stronger exercise attitude in the proximal temporal distance condition, regardless of whether the message was framed in terms of gain or loss. Personal risk perception is also concerned with the health belief (44). When individuals perceive health risks, a strong health belief incentivizes them to accept persuasion, adopt health advice, and avoid the consequences of unhealthy behavior. Prior research on health communication focused on matching the message's framing characteristics with the recipient's health goals. The three-way interaction observed in this study is crucial for advancing our knowledge of the factors that influence adolescents' health behavior. Practitioners of health promotion should create information that is easily understood by adolescents regarding disease risks, thereby increasing adolescents' certainty regarding their existing health conditions.

In addition to these new findings, this study has several limitations that warrant further investigation. First, previous research has shown that message framing, whether framed in terms of gain or loss, positively affects exercise motivation compared to no framing (16). Similar to other research in this field (27, 34), this study did not include a neutral message, so we cannot compare the effect size of the framing effect to that of the no-framing condition. This however should understate the obtained results. Second, this research focused solely on the effects of message framing in the written text category. No comparative cross-sectional analysis of message framing in the audio, image, and video categories was conducted. There is a need for scientific evidence regarding personalized delivery formats for different age groups and sexes in order to optimize the framing effect. Third, there are other moderators and mediators of health message framing besides risk perception. Future research could additionally examine the interactive role of prior exercise experience (45), emotions, self-efficacy, and self-esteem (46), which are expected to serve as mediators between exercise attitude and physical activity.

In conclusion, we found independent effects of message framing and temporal distance on the exercise attitude of Chinese adolescents. In addition, we found that risk perception played an important role in the high-risk group of obesity, suggesting that constructing a message with a proximal temporal distance frame could result in a stronger exercise attitude among the overweight and obese adolescents. These findings contribute theoretically to the existing literature on message framing and provide healthcare practitioners with recommendations for designing youth-tailored messages. Youth health behaviors persist into adulthood, and policymakers in China and around the world should continue to raise public awareness of the growing public health and economic burden of physical inactivity. Tailoring the frame to target populations may be one of the most cost-effective ways to induce behavioral changes for the prevention and treatment of childhood and adolescent obesity.

## Data availability statement

The datasets presented in this study can be found in online repositories. The names of the repository/repositories and accession number(s) can be found below: The data that support the findings of this study are openly available in figshare at https://doi.org/10.6084/m9.figshare.20286075.v2.

## Ethics statement

This experiment was approved by the Ethical Review Board of Hunan Normal University. Written informed consent to participate in this study was provided by the participants' legal guardian/next of kin.

## Author contributions

All authors listed have made a substantial, direct, and intellectual contribution to the work and approved it for publication.

## Funding

This research was funded by the Department of Education of Zhejiang Province: Research on the construction of the carrier for the co-education of civic and political science from the Sanquan Education perspective, Grant Number Y202147685.

## Conflict of interest

The authors declare that the research was conducted in the absence of any commercial or financial relationships that could be construed as a potential conflict of interest.

## Publisher's note

All claims expressed in this article are solely those of the authors and do not necessarily represent those of their affiliated organizations, or those of the publisher, the editors and the reviewers. Any product that may be evaluated in this article, or claim that may be made by its manufacturer, is not guaranteed or endorsed by the publisher.
